# Prognosis of Small Cell Lung Cancer with Idiopathic Pulmonary Fibrosis: Assessment according to GAP Stage

**DOI:** 10.1155/2019/5437390

**Published:** 2019-05-02

**Authors:** Myung Jin Song, Sung Yoon Lim, Jong Sun Park, Ho il Yoon, Jae-Ho Lee, Song Yee Kim, Ji Ye Jung, Young Ae Kang, Moo Suk Park, Young Sam Kim, Joon Chang, Sang Hoon Lee, Choon-Taek Lee

**Affiliations:** ^1^Division of Pulmonology, Department of Internal Medicine, Institute of Chest Diseases, Severance Hospital, Yonsei University College of Medicine, Seoul, Republic of Korea; ^2^Division of Pulmonary and Critical Care Medicine, Department of Internal Medicine, Seoul National University Bundang Hospital, 82 Gumi-ro, 173 Beon-gil, Bundang-gu, Seongnam-si, Gyeonggi-do, 463-707, Republic of Korea

## Abstract

**Introduction:**

Idiopathic pulmonary fibrosis (IPF) is an independent risk factor for lung cancer development, and small cell lung cancer (SCLC) comprises 15-20% of lung cancers with IPF. The objective of this study was to investigate survival outcomes and treatment-related complications according to GAP (gender, age, and physiology) stage in patients having SCLC with IPF (SCLC-IPF).

**Materials and Methods:**

Retrospectively collected data of SCLC-IPF patients from two tertiary care university hospitals in South Korea were reviewed. A total of 59 SCLC-IPF patients were identified and categorized according to GAP stage, which was proposed by Ley et al. in 2012 to predict the prognosis of IPF. Survival outcomes and treatment-related complications were compared between the two groups.

**Results:**

In a total of 59 patients, the median age was 71 years and 58 (98.3%) were male. In a comparison of the median overall survival (OS) according to GAP stage, median OS of the advanced GAP stage group was significantly shorter than median OS of GAP stage I group (7.1 months vs. 16.1 months;* p *= 0.002). Treatment-related complications occurred more frequently in the advanced GAP stage group; advanced GAP stage was the only predictor that exhibited a significant association with the incidence of acute exacerbation of IPF.

**Conclusions:**

Inferior survival outcome and higher incidence of treatment-related pulmonary toxicities were noted in the advanced GAP stage group. Furthermore, advanced GAP stage was the only predictor of treatment-related acute exacerbation of IPF. Physicians should thus consider GAP stage, which reflects the severity of IPF, during treatment of SCLC-IPF.

## 1. Introduction

Idiopathic pulmonary fibrosis (IPF) is the most severe type of idiopathic interstitial pneumonia (IIP), characterized by progressive lung scarring and the histologic pattern of usual interstitial pneumonia (UIP) and affecting approximately 3 million people worldwide [1]. There are several comorbidities associated with IPF development and prognosis, such as chronic obstructive pulmonary disease, emphysema, pulmonary hypertension, gastroesophageal reflux disease, and lung cancer (LC). Notably, among these comorbidities, LC has the most devastating influence on overall outcome of IPF patients [2].

The relative risk of developing LC in IPF is approximately 8 times higher than in the general population [3], and the prevalence of LC in patients with IPF is reported to be 2.7–45.7% [4–8]. Two studies reporting on the cumulative incidence of LC in IPF described concordant increases in incidence as time passed after diagnosis of IPF. Ozawa et al. reported cumulative incidences of 3.3%, 15.4%, and 54.7% of LC in patients with IPF at 1, 5, and 10 years, respectively [7]. Kato et al. reported incidences of 12.2% and 23.3% at 5 and 10 years, respectively [8].

Squamous cell carcinoma is the most common histologic type of LC in patients with IPF, followed by adenocarcinoma, and small cell lung cancer (SCLC) comprises approximately 15-20% of the cases, which is similar to its incidence in the general population [7, 9–11]. SCLC is characterized by rapid doubling time and early development of widespread metastases. It is highly sensitive to initial chemotherapy and radiotherapy, but no effective target agent is known, in contrast to nonsmall cell lung cancer (NSCLC); thus, standard treatments for SCLC remain primarily conventional chemotherapy and radiotherapy. Despite its high sensitivity to chemotherapy and radiotherapy, median overall survival (OS) is disappointing due to the high recurrence rate involved [12]. Because IPF itself has a poor prognosis, a fatal outcome is inevitable when these two diseases are combined. Additionally, SCLC with IPF (SCLC-IPF) patients experience anticancer treatment-related complications more frequently. Previous studies have shown increased chemoradiotherapy-induced pulmonary complications in patients with established pulmonary fibrosis [11, 13, 14]. Anticancer treatment in advanced SCLC-IPF is reported to be somewhat efficient and safe [15–17], but there remains a lack of evidence regarding whether active treatment for LC is truly beneficial.

In 2012, Ley et al. described the GAP (gender, age, and physiology) index and staging system, which can easily be used to estimate mortality in IPF patients. The GAP index includes gender, age, and two lung physiologic variables (forced vital capacity [FVC] and carbon monoxide diffusing capacity [D_LCO_]) [18]. In this study, by using the GAP stage, we aimed to investigate survival outcome and anticancer treatment-related complications in SCLC-IPF.

## 2. Materials and Methods 

### 2.1. Patient Population

**Figure 1:**
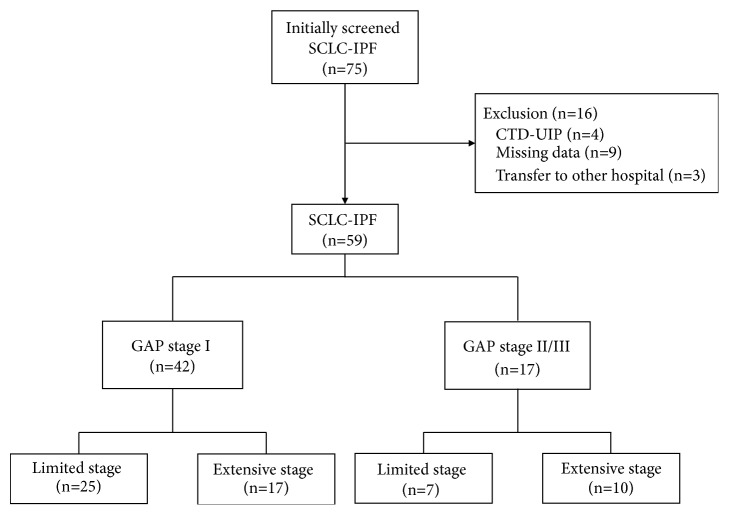
Patient recruitment flow chart. Abbreviations: CTD-UIP, connective tissue disease-related usual interstitial pneumonia; GAP, gender, age, and physiology; SCLC-IPF, small cell lung cancer with idiopathic pulmonary fibrosis.

This study reviewed medical records of two tertiary care university hospitals in South Korea for treatments performed during the indicated periods: Seoul National University Bundang Hospital (November 2003 to March 2018) and Severance Hospital (November 2005 to March 2018). A total of 75 SCLC-IPF patients were initially screened, and 16 were excluded for the following reasons (Figure 1): connective tissue disease-related UIP (n=4), incomplete pulmonary function test results (n=9), and patients transferred to other hospitals without LC treatment (n=3). Finally, 59 SCLC-IPF patients were enrolled. This study was approved by the Institutional Review Board and Ethics Committee of Seoul National University Bundang Hospital (IRB number: B-1707/411-402) and Severance Hospital (IRB number: 4-2018-0432). All methods were performed in accordance with the Declaration of Helsinki.

### 2.2. Definitions

IPF was confirmed via a multidisciplinary approach by pulmonologists, chest specialist radiologist, and pathologists, in accordance with diagnostic criteria defined by the International Consensus Statement of the American Thoracic Society and European Respiratory Society, revised in 2018 [19]. For patients who were suspected of IPF, detailed history taking (including medication, environmental exposure) and serologic tests to exclude connective tissue disease were performed. If there was no potential cause for ILD identified, further diagnostic tests were performed, including chest computed tomography (CT) scan. If the chest CT scan revealed definite UIP pattern, diagnosis of IPF was confirmed. If the chest CT scan revealed probable UIP or indeterminate UIP pattern, the available bronchoalveolar lavage fluid, transbronchial lung biopsy and surgical lung biopsy results were thoroughly reviewed. The decision to diagnose IPF was based on multidisciplinary discussion. Acute exacerbation of IPF was defined as an acute, clinically significant respiratory deterioration, characterized by evidence of new widespread alveolar abnormality. The diagnostic criteria for acute exacerbation of IPF were as follows: previous or concurrent diagnosis of IPF, acute worsening or development of dyspnea in typically less than a month, chest CT with new bilateral ground-glass opacity and/or consolidation superimposed on a background pattern consistent with UIP, and deterioration not fully explained by cardiac failure or fluid overload [20]. The GAP index was calculated using gender (0–1 point), age (0–2 points), FVC (0–2 points), and D_LCO_ (0–3 points); it was categorized into three stages: I (0–3 points), II (4–5 points), and III (6–8 points). GAP stage I, II and III predict 1-year mortality of 6%, 16%, and 39%, respectively, in IPF patients [18]. OS was calculated from the date of LC treatment to the date of death or last follow-up. Tumor response to chemotherapy and concurrent chemoradiotherapy (CCRT) was assessed in accordance with the Response Evaluation Criteria in Solid Tumors (RECIST) criteria, version 1.1 [21]. The objective response rate (ORR) was calculated as the percentage of patients with a complete or partial response according to RECIST criteria.

### 2.3. Statistical Analysis

Continuous variables were analyzed by using the Mann–Whitney U test; categorical variables were analyzed by using the chi-squared distribution and Fisher's exact test. Cumulative time-to event distributions (survival, progression) were estimated by using the Kaplan–Meier method. In all cases, p values < 0.05 were considered statistically significant. All statistical analyses were performed by using IBM SPSS Statistics (version 23.0).

## 3. Results

### 3.1. Patient Characteristics

A total of 59 patients were enrolled; the baseline characteristics of the study population are shown in Table 1. The mean age of study population was 71.0 years (Interquartile range [IQR], 66.0–76.0 years); 58 patients (98.3%) were male and 55 (93.2%) were exposed to smoking. Coexisting emphysema was present in 11 (18.6%) patients. Locations of primary LC were as follows: lower lobe in 26 patients (44.0%), peripheral in 45 (76.3%), and abutting to fibrotic lesion in 43 (72.9%). Thirty-two patients (54.2%) were in limited stage and 27 (45.8%) were in extensive stage at the time of LC diagnosis. By using GAP stage, patients were divided into 3 groups: stage I (n=42), II (n=12), and III (n=5). There were only five patients in the GAP stage III group; we combined GAP stages II and III into an advanced GAP stage group. At baseline, there were no significant differences in age, gender, body mass index (BMI), smoking exposure, or LC staging between GAP stage I and advanced GAP stage groups. In the advanced GAP stage group, patients with high Eastern Cooperative Oncology Group (ECOG) score (score 2 or 3) were more frequent (23.8% vs. 64.7%;* p* = 0.003). Of the 59 total patients, 28 were treated with chemotherapy, 19 with CCRT, six with surgery, and six with conservative care. Of six patients who underwent surgery, two received chemotherapy, two received CCRT as an adjuvant treatment, and two received conservative care without additional anticancer treatment after surgery.

### 3.2. Survival Outcomes

**Figure 2:**
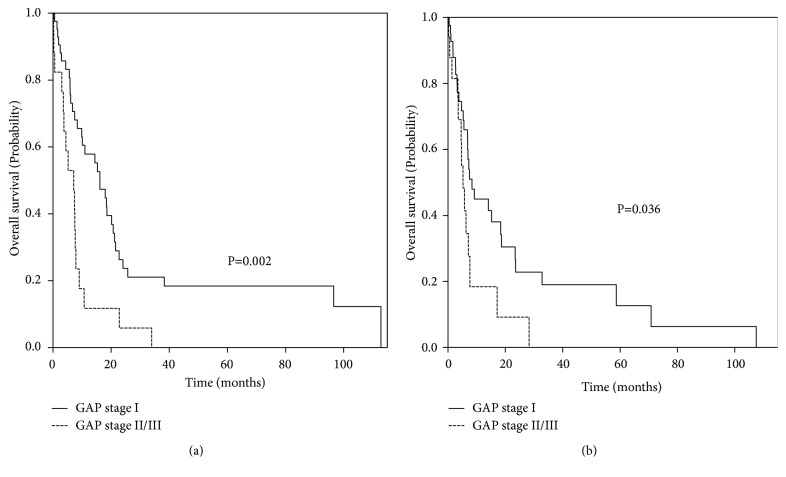
Probability of overall survival and progression-free survival, according to GAP stage. (a) Probability of overall survival, (b) probability of progression-free survival. Abbreviation: GAP, gender, age, and physiology.

Among all 59 patients, median OS and median progression-free survival (PFS) were 9.9 months (IQR, 5.3–21.5) and 7.1 months (IQR, 3.6–18.6), respectively. In a comparison of median OS according to GAP stage, the median OS of the advanced GAP stage group was significantly shorter than that of the GAP stage I group (16.1 months vs. 7.1 months;* p* = 0.002) (Figure 2). Median PFS of the advanced GAP stage group was also significantly shorter than that of the GAP stage I group (8.4 months vs. 5.3 months;* p *= 0.036).

**Figure 3:**
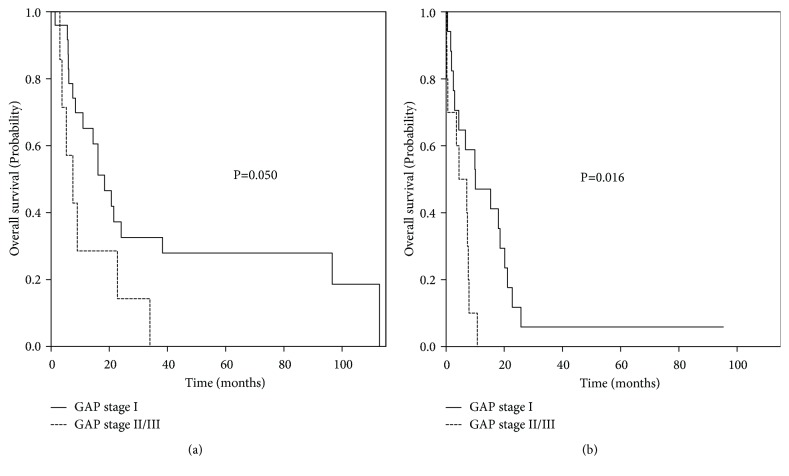
Probability of overall survival, according to GAP stage and SCLC stage. (a) Survival probability of limited stage, (b) survival probability of extensive stage. Abbreviations: GAP, gender, age, and physiology; SCLC, small cell lung cancer.

Subgroup analysis of survival outcomes was conducted by dividing cases into limited and extensive stage disease. In extensive stage SCLC, median OS of the advanced GAP stage group was significantly shorter than that of the GAP stage I group (10.1 months vs. 4.5 months;* p* = 0.016). In limited stage SCLC, median OS of the advanced GAP stage group was also shorter than that of the GAP stage I group; however, this was not statistically significant (18.4 months vs. 7.5 months;* p* = 0.050) (Figure 3).

### 3.3. Response to Chemotherapy and CCRT

The ORR of 28 patients who received chemotherapy as a first-line treatment was 64.3%. There was no significant difference in the ORR of chemotherapy between GAP stage I and advanced GAP stage groups (63.2% vs. 66.7%;* p* = 1.000). The ORR of 19 patients who received CCRT as a first-line treatment was 84.2%. There was also no significant difference in the ORR of CCRT between GAP stage I and advanced GAP stage groups (81.4% vs. 60.0%;* p* = 0.155).

### 3.4. Treatment-Related Complications

To assess the incidence of complications related to anticancer treatment, the duration between last treatment and onset of complication event was defined as 4 weeks or less. Six patients who received best supportive care for SCLC were excluded from treatment-related complication analysis. Overall complications, regardless of treatment modality, are shown in Table 2. Acute exacerbation of IPF and pneumonia occurred in 16 (30.2%) and 18 (34.0%) patients, respectively. According to GAP stage, acute exacerbation of IPF and pneumonia occurred more frequently in the advanced GAP stage group (20.5% vs. 57.1%,* p *= 0.017; 25.6% vs. 57.1%,* p *= 0.049). Cytopenia, gastrointestinal trouble, and pulmonary thromboembolism did not differ significantly between the GAP stage groups.

Subgroup analysis of complications according to treatment modality is shown in Table 3. As mentioned above, among the 6 patients who underwent surgery, 2 received chemotherapy and 2 received CCRT after surgery. Complication events that occurred within 4 weeks after surgery or before the initiation of chemotherapy or CCRT were included as surgery-related complications, and complications that occurred at 4 weeks after surgery or after the initiation of adjuvant chemotherapy and CCRT were classified as chemotherapy and CCRT-related complications.

In patients who received chemotherapy, the incidence of acute exacerbation of IPF was significantly higher in the advanced GAP stage group than in the GAP stage I group (14.3% vs. 66.7%;* p *= 0.008). Other complications including pneumonia showed no significant difference between the GAP stage I and advanced GAP stage groups.

In patients who received CCRT, the incidences of pneumonia and radiation pneumonitis were significantly higher in the advanced GAP stage group than in GAP stage I group (6.3% vs. 80.0%, p=0.004; 25.0% vs. 80.0%,* p* = 0.047), although there was no significant difference in radiation modality and dose.

### 3.5. Predictors of Acute Exacerbation of IPF

Logistic regression analysis of individual variables for the acute exacerbation of IPF is shown in Table 4. Advanced GAP stage was the only predictor that exhibited a significant association with the incidence of acute exacerbation of IPF (adjusted hazard ratio, 5.851; 95% confidential interval, 1.453–23.565;* p* = 0.013). Amount of smoking exposure (pack-years) tended to be related to the incidence of acute exacerbation of IPF but was not statistically significant.

## 4. Discussion

In this study, we demonstrated survival outcomes and treatment-related complications of SCLC-IPF according GAP staging system, which reflects the severity of IPF and predicts mortality. Patients with advanced GAP stage showed an inferior survival outcome and higher incidence of treatment-related pulmonary toxicities than those with GAP stage I, and advanced GAP stage was the only predictor of treatment-related acute exacerbation of SCLC-IPF. To our knowledge, there has been no previous report of survival outcome and complications in SCLC-IPF patients, according to GAP stage.

LC is commonly comorbid in IPF patients, exhibiting 6.6% prevalence among IPF patients in a Korean national survey [22]. The significance of the combination of these two diseases is receiving increased attention with the emerging evidence of pathogenic mechanisms linking LC and IPF [13, 23, 24]. However, standard treatment for LC with IPF has not yet been established. Compared to NSCLC, there is little information regarding the prognosis and treatment-related complications of SCLC-IPF. In this study, we investigated survival outcomes and anticancer treatment-related complications in SCLC-IPF patients according to GAP stage, which represents the severity and prognosis of IPF.

In the general population, the median OS of SCLC are 18–24 months and 9–10 months for patients with limited stage and extensive stage, respectively [12]. In this study, the median OS of GAP stage I group were 18.4 months (IQR, 7.5–96.6) for limited stage SCLC and 10.1 months (IQR, 2.9–20.2) for extensive stage SCLC. This result suggests that survival outcome in the GAP stage I group is equivalent to that observed previously in the general population, despite the combination of two devastating diseases. Unlike the GAP stage I group, patients with advanced GAP stage showed inferior survival outcomes than the general population, in both limited stage and extensive stage.

In previous studies of SCLC with IIP, the survival outcome of extensive stage SCLC was reported to be comparable to that of extensive stage SCLC without IIP, but the survival outcome of limited stage SCLC was inferior to that of limited stage SCLC without IIP [15, 16, 25, 26]. Two of these studies did not include patients' lung physiologic variables [25, 26], and two other studies implied FVC as a result of demographic data [16, 25]. Mean ± standard deviations of FVC % predicted reported in these studies were 91.4 ± 16.9 and 89.9 ± 14.4, respectively, which suggest that relatively mild IIP patients were enrolled. Except for one study that limited interstitial pneumonia to IPF [25], the definition used to define IIP differed among the studies. Because IIP is a heterogeneous disease entity that has several different clinical–radiologic–pathologic diagnoses, including IPF and each with a different prognosis and clinical features, we believe it is necessary to evaluate survival outcome of SCLC in the IPF patients group specifically, not in all patients with IIP. In this study, we analyzed survival outcomes of SCLC-IPF according to GAP stage, which includes lung physiologic variables, and revealed inferior survival outcome in the advanced GAP stage group, regardless of LC stage.

The incidences of treatment-related pulmonary toxicities, such as acute exacerbation of IPF and pneumonia, were significantly higher in the advanced GAP stage group. When divided according to treatment modalities, the incidence of acute exacerbation of IPF was significantly different between GAP stage I and advanced GAP stage groups in patients who received chemotherapy, but not in patients who received CCRT. This might be due to different compositions of LC stage between the two different treatment modality groups. In the CCRT group, all patients were in limited stage; in the chemotherapy group, the majority of patients were in extensive stage (73.3%). This result suggests that the GAP stage is more useful to predict the incidence of acute exacerbation of IPF in extensive stage.

Previous studies reported the incidence of acute exacerbation in SCLC with IIP to be 11.9–36.4%; however, no studies revealed meaningful predictors for acute exacerbation [16, 25, 26]. This might be due to small sample sizes and heterogeneous characteristics of IIP. The present study revealed an incidence of 30.2% for acute exacerbation after anticancer treatment, consistent with previous studies. Logistic regression analysis to reveal predictors for acute exacerbation in SCLC-IPF showed that advanced GAP stage was the only statistically meaningful predictor. Several studies have analyzed the incidence of acute exacerbation in overall LC patients or in NSCLC patients. Those studies reported that the UIP pattern of interstitial pneumonia and low FVC were predictors for treatment-related acute exacerbation of interstitial pneumonia [14, 27]. There is a series of studies which applied modified GAP index to NSCLC with ILD patients [28, 29]. The modified GAP index includes sex, age, and FVC; it does not include D_LCO_ because of a lack of data. The study revealed that modified GAP index was associated with the incidence of ILD acute exacerbation in NSCLC with ILD patients. Unlike the prior studies, our study used the original GAP index, including D_LCO_, and validated the GAP index for SCLC-IPF.

Regarding the chemotherapy agent, platinum-based chemotherapy is the most commonly used standard first-line chemotherapy. In our study, a majority of patients were treated with etoposide + carboplatin or cisplatin combinations; the chemotherapy regimens used in our study are summarized in Table 5. This study included chemotherapy agents only for first-line treatment. Since the survival outcome of GAP stage I group was equivalent to that of the general population group, further studies are needed to evaluate the efficacy of chemotherapy agents used for recurrent SCLC with preexisting IIP patients.

Despite the lethal treatment-related complications, there is no specific treatment guideline established for LC with IPF. Hence, for the physician, it is crucial to discriminate patients who would be beneficial to the cancer treatment from patients who are at high risk of treatment-related complications. We expect this study to provide treatment guidance to physicians who are unsure whether to initiate anticancer treatment in SCLC-IPF patients. Physicians can attempt active anticancer treatment in patients with GAP stage I and should be more cautious of treatment-related complications in patients with advanced GAP stages.

There are promising areas in the therapeutic approach of LC with IPF when antifibrotic agents, pirfenidone and nintedanib which are approved for treatment of IPF, are combined with conventional cancer treatment [30]. Regarding pirfenidone, its prophylactic effect for postoperative acute exacerbation of IPF in LC was demonstrated by recently published studies [31, 32]. Moreover, nintedanib was initially approved for treatment of NSCLC in combination with docetaxel-based second-line therapy [33], but further clinical trials are required to evaluate the effect of these antifibrotic agents in patients with IPF in LC.

This study has several limitations. First, this was a retrospective nonrandomized study, in which there can be various biases and confounding factors. However, we obtained data from two tertiary care hospitals to reduce biases. Second, only pathologically confirmed SCLC patients were enrolled in the study; thus, patients whose disease was too severe to complete the diagnostic procedure might have been excluded. Third, patients who did not undergo pulmonary function test and patients who were transferred to other hospitals without anticancer treatment were excluded from this study. This selection bias may have influenced the final results. Further studies are needed to validate the result in larger sample groups.

## 5. Conclusions

We found an inferior survival outcome and higher incidence of treatment-related pulmonary toxicities in the advanced GAP stage group. Advanced GAP stage was a predictor of treatment-related acute exacerbation of IPF. This result can provide guidance to distinguish patients who would tolerate anticancer treatment from patients who are at high risk of treatment-related lethal complications.

## Figures and Tables

**Table 1:** Baseline characteristics, according to GAP stage.

	Total (n=59)	GAP stage I (n=42)	GAP stage II/III (n=17)	p value
Age, median (IQR)	71.0 (66.0, 76.0)	70.0 (64.8, 75.3)	71.0 (66.5, 76.0)	0.150
Male, No. (%)	58 (98.3)	41 (97.6)	17 (100)	0.521
BMI, median (IQR), kg/m2	23.2 (21.8, 25.6)	23.2(21.7, 25.6)	23.3(21.3, 25.9)	0.426
Smoking exposure, No. (%)				
Never	4 (6.8)	2 (4.8)	2 (11.8)	0.084
Former	34 (57.6)	28 (66.7)	6 (35.3)	
Current	21 (35.6)	12 (28.6)	9 (52.9)	
Pack-years, median (IQR)	40.0 (25.0, 50.0)	40.0 (25.0, 50.0)	41.0 (30.0, 50.0)	0.542
Emphysema, No. (%)	11 (18.6)	7 (16.7)	4 (23.5)	0.540
ECOG score, No. (%)				0.003
0, 1	38 (64.4)	32 (76.2)	6 (35.3)	
2, 3	21 (35.6)	10 (23.8)	11 (64.7)	
FVC % pred, median (IQR)	83.0 (68.0, 93.0)	86.0 (80.0, 95.3)	66.0 (61.5, 79.0)	<0.001
D_LCO_ % pred, median (IQR)	75.5 (65.0, 85.0)	80.5 (68.5, 85.0)	66.0 (54.3, 75.0)	0.039
Location, No. (%)				0.856
Right upper lobe	5 (8.5)	3 (7.1)	2 (11.8)	
Right middle lobe	11 (18.6)	7 (16.7)	4 (23.5)	
Right lower lobe	12 (20.3)	8 (19.0)	4 (23.5)	
Left upper lobe	17 (28.8)	13 (31.0)	4 (23.5)	
Left lower lobe	14 (23.7)	11 (26.2)	3 (17.6)	
Abutting to fibrotic lesion, No. (%)	43 (72.9)	32 (76.2)	11 (64.7)	0.369
Peripheral, No. (%)	45 (76.3)	33 (78.6)	12 (70.6)	0.514
Stage, No. (%)				0.254
Limited stage	32 (54.2)	25 (59.5)	7 (41.2)	
Extensive stage	27 (45.8)	17 (40.5)	10 (58.8)	
Primary treatment, No. (%)				0.269
Conservative care	6 (10.2)	3 (7.1)	3 (17.6)	
Surgery	6 (10.2)	6 (14.3)	0 (0.0)	
Chemotherapy	28 (47.5)	19 (45.2)	9 (52.9)	
Concurrent chemoradiotherapy	19 (32.2)	14 (13.3)	5 (29.4)	
Median OS, median (IQR), months	9.9 (5.3-21.5)	16.1 (10.8-21.4)	7.1 (3.2-11.1)	0.002
Median PFS, median (IQR), months	7.1 (3.6-18.6)	8.4 (5.3-11.4)	5.3 (3.4-7.1)	0.036

Abbreviations: BMI, body mass index; D_LCO_, carbon monoxide diffusing capacity; ECOG, Eastern Cooperative Oncology Group; FVC, forced vital capacity; GAP, gender, age, and physiology; IQR, interquartile range; OS, overall survival; PFS, progression-free survival.

**Table 2:** Anticancer treatment-related complications, classified according to GAP stage.

	Total (n=53)	GAP stage I (n=39)	GAP stage II/III (n=14)	p value
AE-IPF	16 (30.2)	8 (20.5)	8 (57.1)	0.017
Pneumonia	18 (34.0)	10 (25.6)	8 (57.1)	0.049
Cytopenia	32 (60.4)	23 (59.0)	9 (64.3)	0.727
GI trouble	18 (34.0)	11 (28.2)	7 (50.0)	0.191
PTE	5 (9.4)	2 (5.1)	3 (21.4)	0.108

Values are presented as No. (%).

Abbreviations: AE-IPF, acute exacerbation of idiopathic pulmonary fibrosis; GAP, gender, age, and physiology; GI, gastrointestinal; PTE, pulmonary thromboembolism.

**Table 3:** Anticancer treatment-related complications, classified according to GAP stage and treatment modality.

	Total	GAP stage I	GAP stage II/III	p value
Surgery (Total=6, GAP stage I=6, GAP stage II/III =0)				
AE-IPF	1 (16.7)	1 (16.7)	0	
Pneumonia	2 (33.3)	2 (33.3)	0	
Pneumothorax	2 (33.3)	2 (33.3)	0	
Chylothorax	1 (16.7)	1 (16.7)	0	
MV > 2 days	1 (16.7)	1 (16.7)	0	

Chemotherapy (Total=30, GAP stage I=21, GAP stage II/III =9)				
AE-IPF	9 (31.0)	3 (14.3)	6 (66.7)	0.008
Pneumonia	11 (36.7.0)	7 (33.3)	4 (44.4)	0.687
Cytopenia	17 (56.7)	12 (57.1)	5 (55.6)	1.000
GI trouble	13 (43.3)	8 (38.1)	5 (55.6)	0.443
PTE	4 (13.3)	1 (4.8)	3 (33.3)	0.069
Neuropathy	3 (10.3)	2 (9.5)	1 (11.1)	1.000

Concurrent chemoradiotherapy (Total=21, GAP stage I=16, GAP stage II/III =5)				
AE-IPF	6 (28.6)	4 (25.0)	2 (40.0)	0.598
Pneumonia	5 (22.7)	1 (6.3)	4 (80.0)	0.004
Cytopenia	15 (71.4)	11 (68.8)	4 (80.0)	1.000
GI trouble	5 (23.8)	3 (18.8)	2 (40.0)	0.553
PTE	1 (4.8)	1 (6.3)	0 (0.0)	1.000
Pneumonitis	8 (36.4)	4 (25.0)	4 (80.0)	0.047
Esophagitis	3 (14.3)	2 (12.5)	1 (20.0)	1.000

Values are presented as No. (%).

Abbreviations: AE-IPF, acute exacerbation of idiopathic pulmonary fibrosis; GAP, gender, age, and physiology; GI, gastrointestinal; MV, mechanical ventilation; PTE, pulmonary thromboembolism.

**Table:** (a) Univariate logistic regression analysis

	HR	95%CI	p value
Age	1.064	0.980-1.155	0.137
ECOG (0-1 vs. 2-3)	1.620	0.466-5.628	0.448
FVC % pred	0.961	0.900-1.026	0.236
GAP stage (Stage I vs. Stage II/III)	5.167	1.390-19.210	0.014
SCLC stage (Limited vs. Extensive)	2.374	0.718-7.852	0.157
Smoking, Pack-years	1.026	0.997-1.055	0.081
Emphysema	0.612	0.112-3.334	0.570

**Table:** (b) Multivariate logistic regression analysis

	HR	95%CI	p value
GAP stage (Stage I vs. Stage II/III)	5.851	1.453-23.565	0.013
SCLC stage (Limited vs. Extensive)	1.948	0.510-7.442	0.329
Smoking, Pack-years	1.028	0.998-1.060	0.066
Emphysema	0.345	0.043-2.766	0.316

Abbreviations: ECOG, Eastern Cooperative Oncology Group; FVC, forced vital capacity; GAP, gender, age, and physiology; IPF, idiopathic pulmonary fibrosis; SCLC, small cell lung cancer.

**Table 5:** Chemotherapy regimens in this study.

	Total	GAP stage I	GAP stage II/III
Chemotherapy (Total=30, GAP stage I=21, GAP stage II/III =9)			
EC	11 (36.7)	7 (33.3)	4 (44.4)
EP	13 (43.3)	12 (57.1)	1 (11.1)
IP	4 (13.3)	1 (4.8)	3 (33.3)
BP	2 (6.7)	1 (4.8)	1 (11.1)

Concurrent chemoradiotherapy (Total=21, GAP stage I=16, GAP stage II/III=5)			
EC	8 (38.1)	6 (37.5)	2 (40.0)
EP	10 (47.6)	8 (50.0)	2 (40.0)
IP	3 (14.3)	2 (12.5)	1 (20.0)

Values are presented as No. (%).

Abbreviations: BP, belotecan+cisplatin; EC, etoposide+carboplatin; EP, etoposide+cisplatin; IP, irinotecan+cisplatin.

## Data Availability

The data used to support the findings of this study are available from the corresponding author upon request.
